# Clinical Insights From an 11‐Year and 6‐Month Follow‐Up of a Full‐Mouth Lithium Disilicate Rehabilitation for Severe Tooth Wear

**DOI:** 10.1111/jerd.70103

**Published:** 2026-01-09

**Authors:** José Maurício dos Santos Nunes Reis, Filipe de Oliveira Abi‐Rached, Marcelo Antonialli Del'Acqua, Bruno Arruda Mascaro, Luis Felipe Rondón

**Affiliations:** ^1^ Department of Dental Materials and Prosthodontics São Paulo State University (UNESP), Araraquara School of Dentistry Araraquara Sao Paulo Brazil; ^2^ São Paulo Association of Dental Surgeons (APCD) Araraquara Sao Paulo Brazil

**Keywords:** early oral aging syndrome, full‐mouth rehabilitation, lithium disilicate, long‐term follow‐up, tooth wear

## Abstract

**Objective:**

This article aims to describe the clinical execution and management of a full‐mouth rehabilitation in a patient with severe tooth wear, utilizing partial and full‐contour lithium disilicate restorations, with a long‐term follow‐up of 11 years and 6 months.

**Clinical Considerations:**

Over a follow‐up of more than 11 years, this case provides in‐depth clinical insight into identifying etiological factors, reestablishing the vertical dimension of occlusion, and implementing lithium disilicate restorations in both partial‐ and full‐contour designs. It also addresses the management of underlying conditions and outlines the challenges encountered during long‐term follow‐up of a full‐mouth rehabilitation for severe tooth wear.

**Conclusions:**

The full‐mouth rehabilitation using partial and full‐contour lithium disilicate restorations for severe tooth wear demonstrated a favorable outcome. At 11+‐year follow‐up, the case exhibited appropriate mechanical behavior, preserved esthetic outcomes, and functional integrity, addressing the complex multifactorial etiological factors associated with tooth wear.

**Clinical Significance:**

Long‐term success in rehabilitating patients with severe tooth wear relies not only on appropriately indicated and executed restorative treatment but also on an interdisciplinary approach that includes effective management of underlying etiological factors and parafunctional habits.

## 1 Introduction

In recent decades, tooth wear has become increasingly prevalent, affecting not only the elderly but also younger and working‐age individuals [1, 2, 3, 4]. Clinically, it is characterized by the loss of dental hard tissues through non‐carious lesions such as attrition, abrasion, abfraction, and erosion—the latter more accurately referred to as biocorrosion [3, 4, 5]. As a result, patients may present an oral condition indicative of a state that does not align with their chronological age [6]. This multifactorial condition has recently been described as early oral aging syndrome [6, 7] and is associated with gastroesophageal disorders [8, 9], psychological conditions [10], and dietary habits [4], involving factors that extend beyond the scope of dentistry but must be effectively managed to achieve successful treatment outcomes [3].

Tooth wear progressively compromises both the appearance and function of patients, potentially leading to alterations in vertical dimension of occlusion (VDO) [11, 12] if not diagnosed in time, making restorative and prosthetic intervention necessary [13, 14]. Thus, direct [15], indirect [16], and combined [17, 18, 19, 20] restorative approaches have been described in the literature, employing resin‐based composites [21] or ceramics [22], depending mainly on the amount of tooth wear. These systemic and oral conditions affect not only the dental structures but also restorative materials [23]. Both resin‐based composites [24, 25] and ceramics [26, 27] undergo significant degradation in their mechanical and optical properties, as well as in surface characteristics, when exposed to acidic environments.

When VDO should be reestablished due to severe tooth wear and inadequate existing restorations in the posterior region, along with compromised buccal and lingual surfaces of the anterior teeth, indirect ceramic restorations are commonly the preferred option because of their mechanical performance and esthetic advantages [14, 28, 29]. Among ceramics, lithium disilicate restorations are the most frequently reported material in this type of clinical scenario, showing a high long‐term survival rate (> 6 years) [29].

Clinical prospective [30, 31, 32, 33, 34, 35] and retrospective [36] trials have consistently reported the favorable performance of this ceramic material in the management of severe tooth wear, including anterior and posterior, full‐contour, and partial restorations, demonstrating an overall survival rate of 98.6% with a 14.1‐year follow‐up [30]. Similarly, survival rates of 98.4% for posterior and 100% for anterior restorations have been reported with a mean 12‐year follow‐up [31]. A total failure rate of 5.5% over a mean observation period of 8.5 ± 2.7 years has also been documented [32]. Regarding crowns specifically, a success rate of 98.6% over a 6‐year period has been reported [33]. Additionally, a retrospective study with a 6 ± 2.4‐year follow‐up showed a 100% survival rate and a 0% complication rate [36]. Focusing on the posterior region, pressed overlays demonstrated a 100% survival rate after a mean follow‐up of 7.9 years [34], while computer‐aided design and computer‐aided manufacturing (CAD/CAM) occlusal veneers showed a survival rate of 88.4% up to 3 years, with lithium disilicate performing slightly better than resin‐based composites [35].

As described, this restorative approach demonstrates favorable performance. Although the choice of material or technique is an important consideration, when appropriately indicated and executed, the management of additional factors—such as gastroesophageal disorders, psychological conditions, dietary habits, and parafunctional behaviors—may be even more critical to the functional and esthetic success of the treatment, as well as to the patient's overall health [6]. This article provides a detailed description of the execution and management of a full‐mouth rehabilitation in a patient with severe tooth wear, using lithium disilicate partial and full‐contour restorations, with a long‐term follow‐up of 11 years and 6 months.

## 2 Clinical Reports

A 47‐year‐old male patient attended a dental office with complaints related to anterior esthetics, hypersensitivity, and posterior diastemas between all the second premolar and first molar following orthodontic treatment. The patient exhibited a tendency toward Class III malocclusion prior to undergoing orthodontic therapy. After communication with the orthodontist, the oral rehabilitator was consulted to determine whether the diastemas could be closed through restorative treatment, as achieving this outcome through additional orthodontic intervention was deemed unfeasible.

**FIGURE 1:**
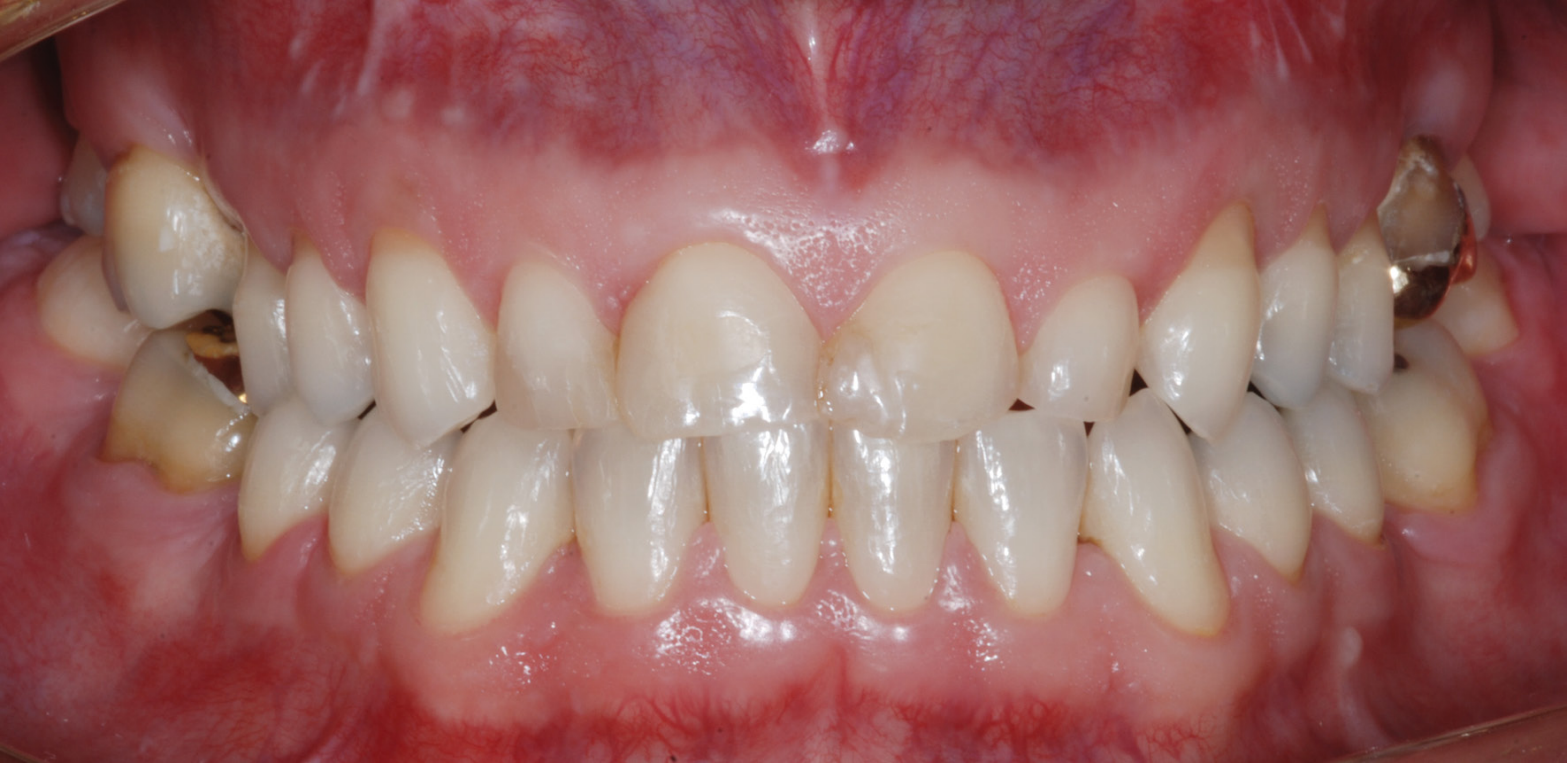
Intraoral frontal view of the preoperative condition showing various patterns of tooth wear, non‐carious cervical lesions, and defective resin‐based composite restorations.

**FIGURE 2:**
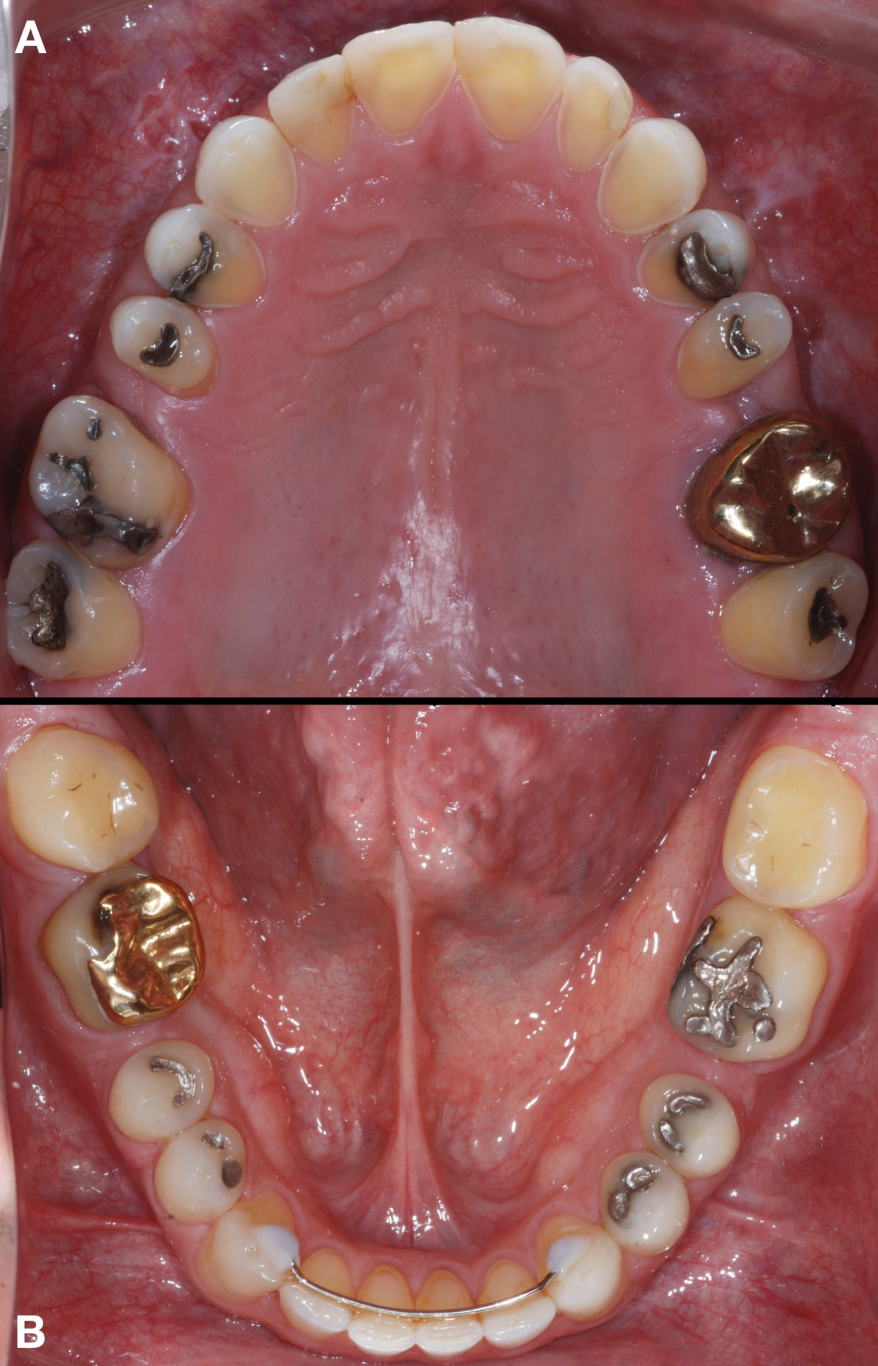
(A) Occlusal view of the maxillary arch showing the biocorrosion pattern on the palatal surfaces, as well as protruding amalgam restorations and a gold crown restoration. (B) Occlusal view of the mandibular arch showing tooth wear, protruding amalgam restorations, and a gold overlay restoration.

During the clinical examination, different patterns of tooth wear—attrition, abrasion, and biocorrosion—were observed, involving the buccal, incisal, occlusal, and particularly the palatal surfaces of the upper arch teeth, with biocorrosion being the most pronounced. Defective anterior resin‐based composite restorations and protruding posterior amalgam restorations were also noted, along with existing gold crown and onlay restorations. The VDO was compromised, and non‐carious cervical lesions were observed on canines and posterior teeth, associated with gingival recession (Figures 1 and 2). The patient's clinical history revealed contributing factors to the severe tooth wear, including gastroesophageal reflux disease, bulimia, psychological disorders, and sleep bruxism.

**FIGURE 3:**
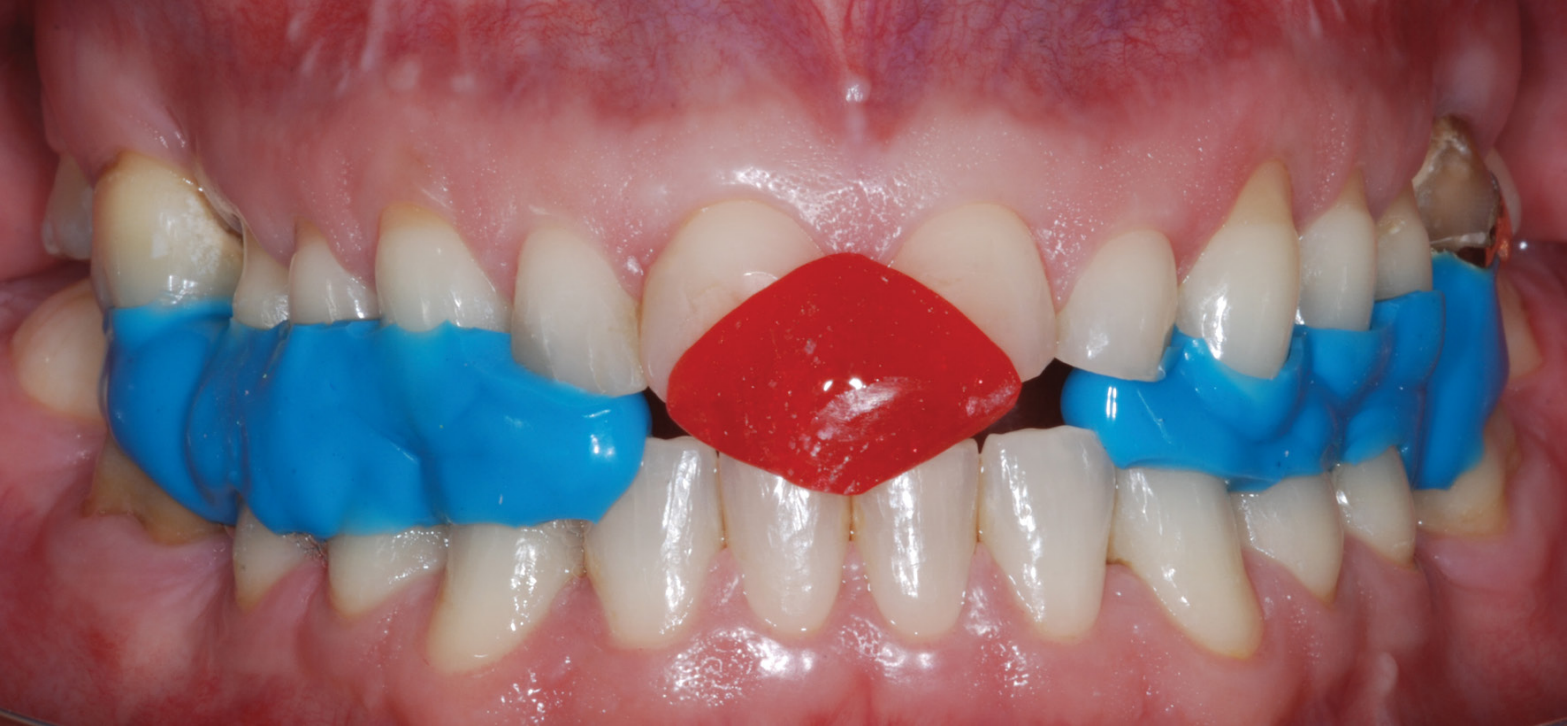
Lucia's JIG and silicone registration used to record the reestablished vertical dimension of occlusion. These apparatus were used to mount the dental casts in a semi‐adjustable articulator for a full‐mouth additive diagnostic wax‐up.

**FIGURE 4:**
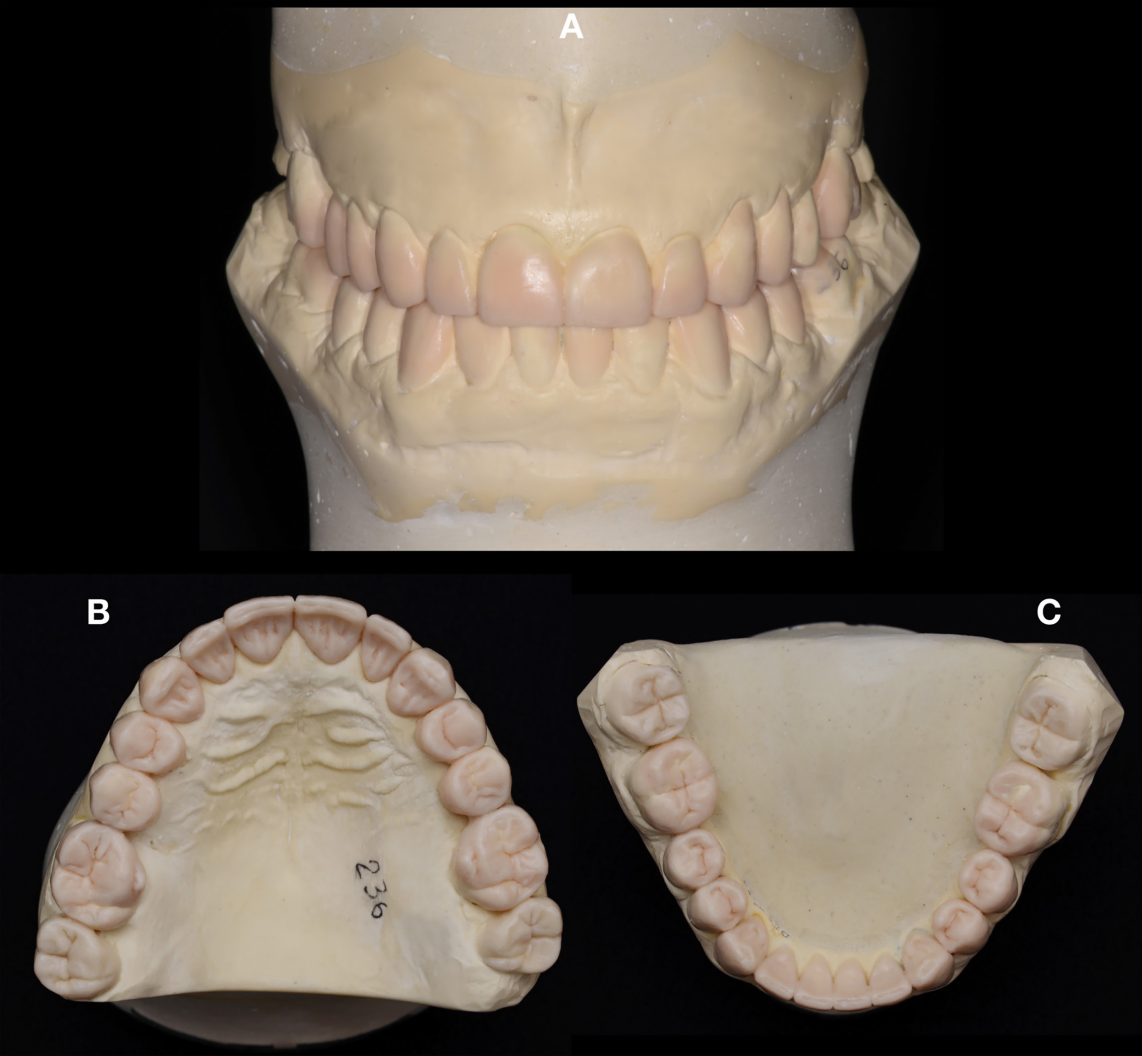
Dental casts mounted in the articulator with the full‐mouth additive wax‐up. (A) Frontal view. (B) Maxillary occlusal view. (C) Mandibular occlusal view.

For the restorative treatment, partial and full‐contour lithium disilicate restorations were proposed, in conjunction with the management of the related systemic conditions by the respective medical specialists. Following informed consent, the patient agreed to treatment, photographic records, and scientific publication of his case. The first clinical stage involved treatment planning. To reestablish the VDO, the Lucia jaw interference guide (JIG) [37] and silicone registration (Regular body—Express XT, 3M ESPE, Saint Paul, MN, USA) were used and then transferred to an articulator with dental casts for a full‐mouth additive wax‐up (Figures 3 and 4). The patient was positioned in centric relation, and esthetic, phonetic, proportion, and functional tests were performed. This process enabled correction of the smile line and the curves of Wilson and Spee, thereby restoring both esthetics and function.

**FIGURE 5:**
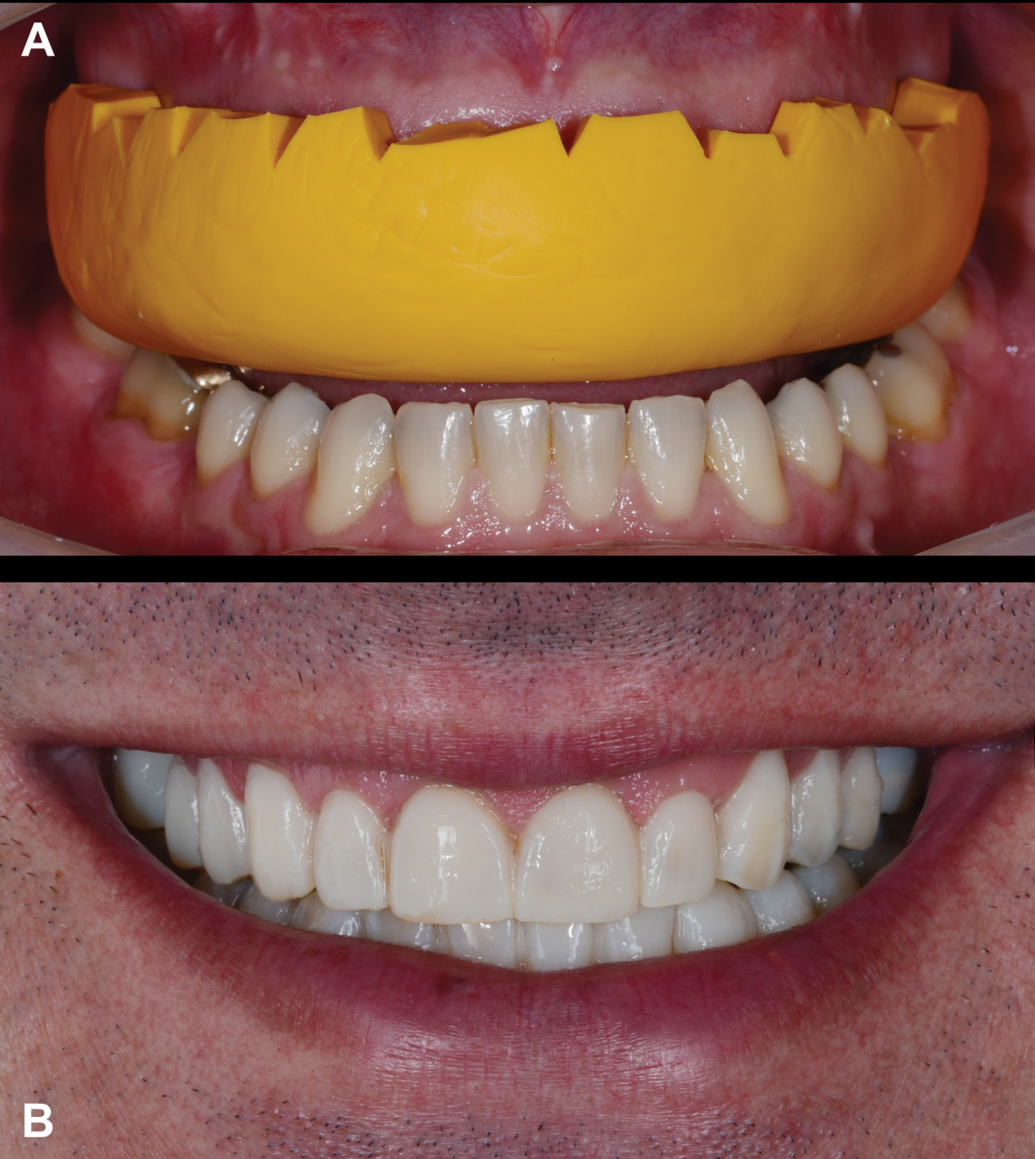
(A) Silicone index in place for testing the intraoral diagnostic mock‐up. (B) Mock‐up positioned to assess the planned restorations.

A diagnostic mock‐up (Protemp 4, 3M ESPE, Seefeld, Germany) was used to assess the esthetic and functional aspects of the planned restorations by both the dental team and the patient (Figure 5). The treatment plan was approved, with the exception of any treatment for the lower anterior teeth. Overlays were recommended for the lower posterior teeth. In the upper arch, crowns were planned for the incisors, canines, premolars, and the left first molar (to replace a previous gold crown), while overlays were indicated for the remaining molars.

**FIGURE 6:**
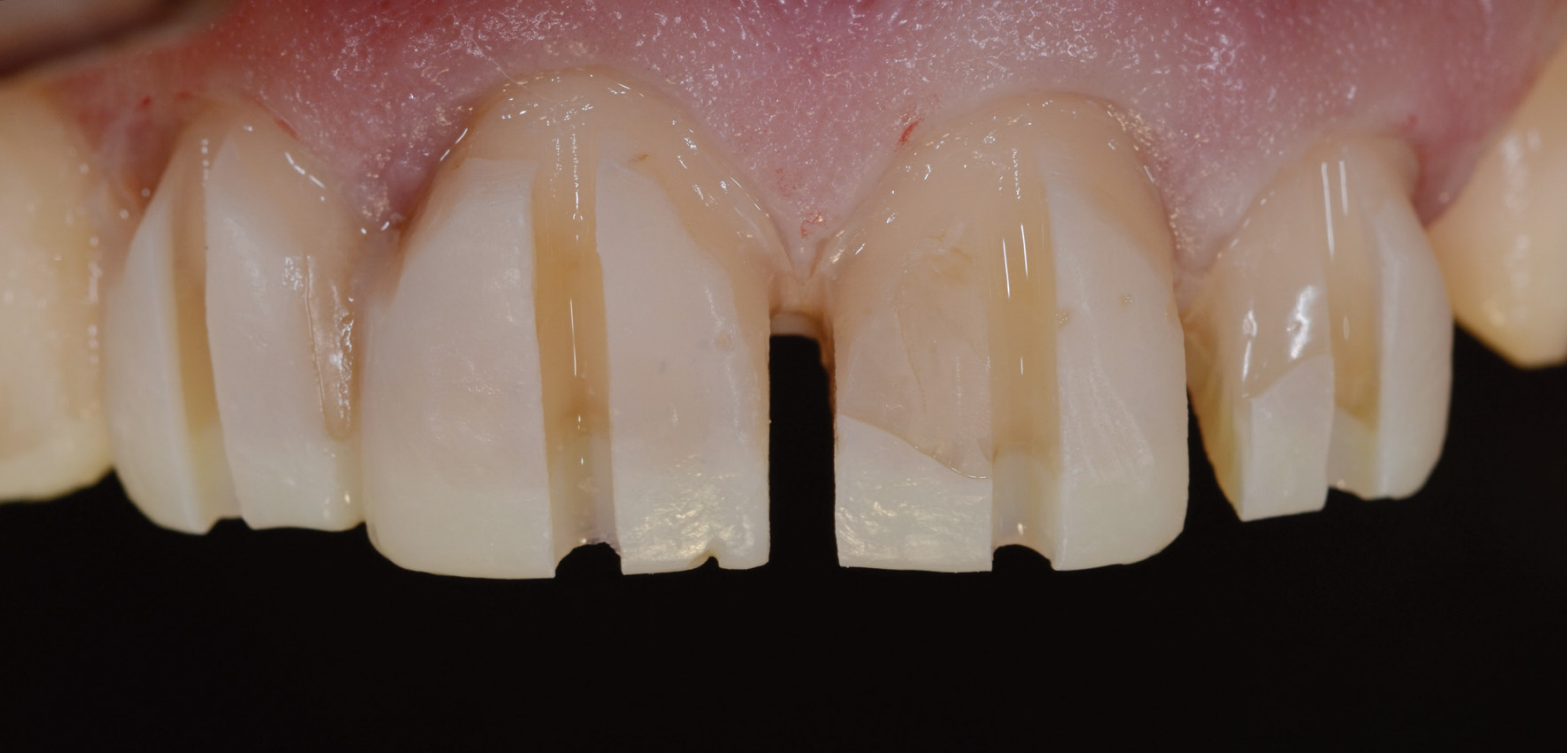
Tooth preparation using the mock‐up as a guide.

**FIGURE 7:**
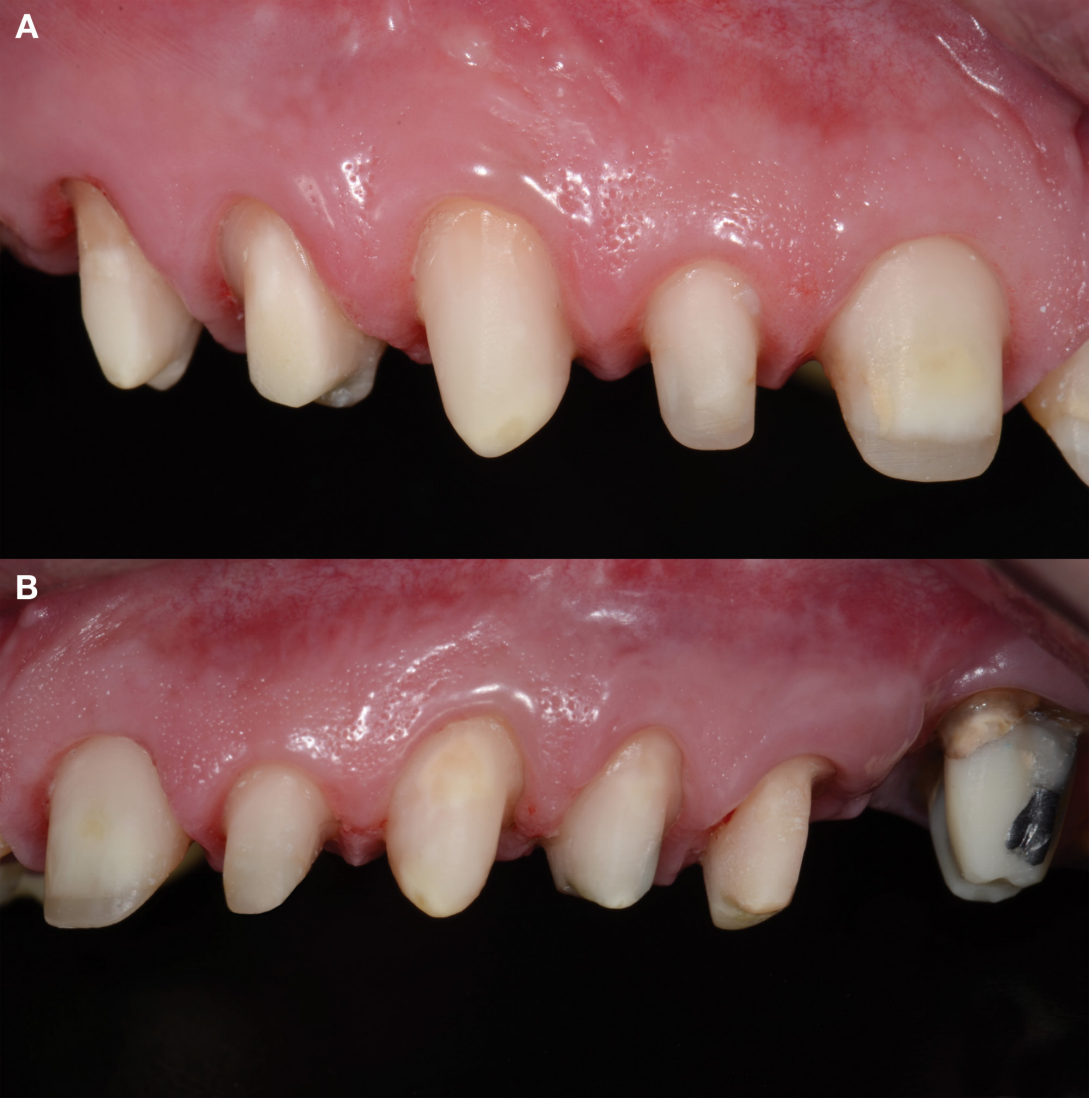
(A) Right lateral view showing crown preparations. (B) Left lateral view showing crown preparations.

**FIGURE 8:**
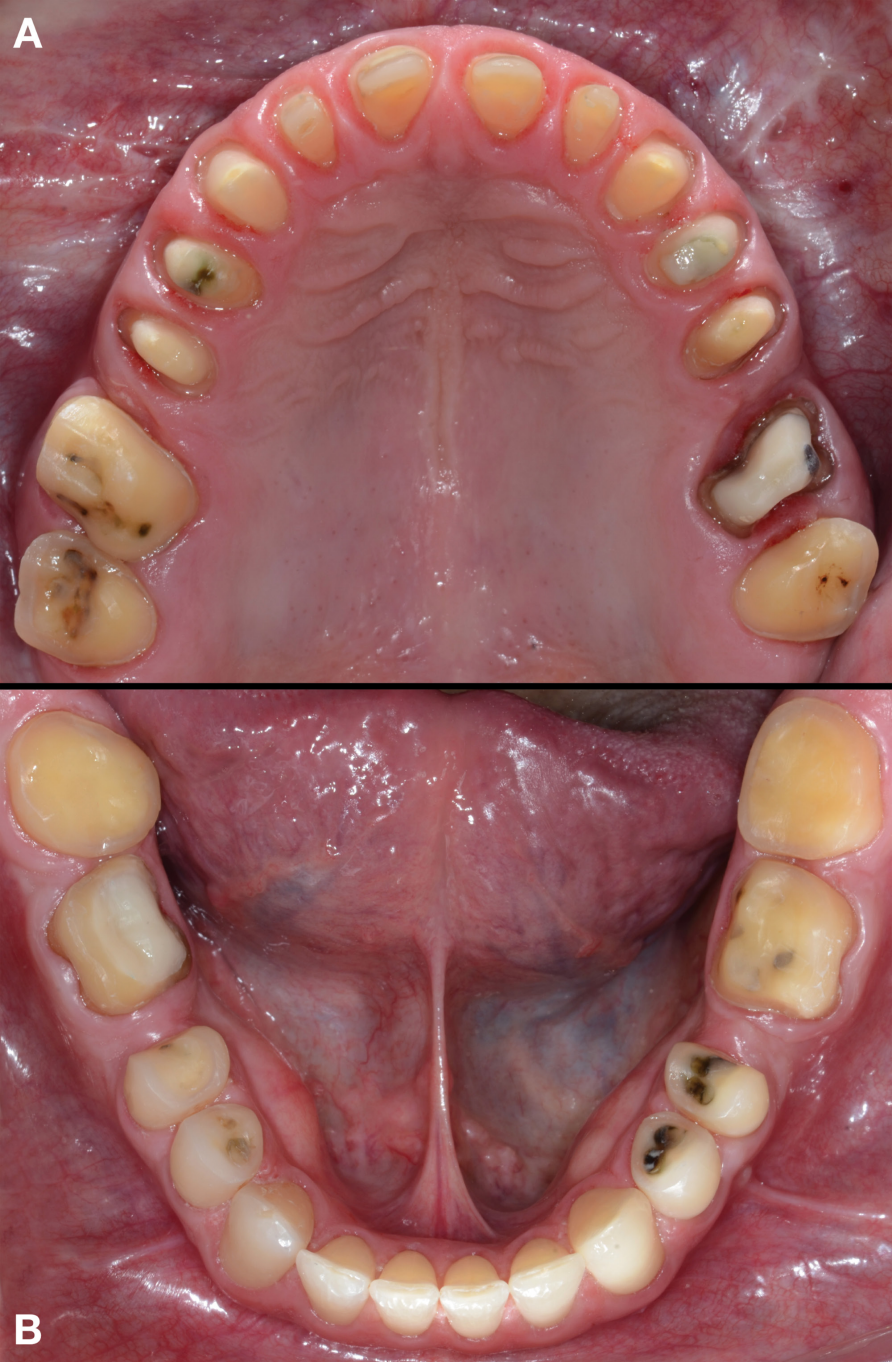
(A) Occlusal view of the dental preparations in the maxillary arch. (B) Occlusal view of the dental preparations in the mandibular arch.

**FIGURE 9:**
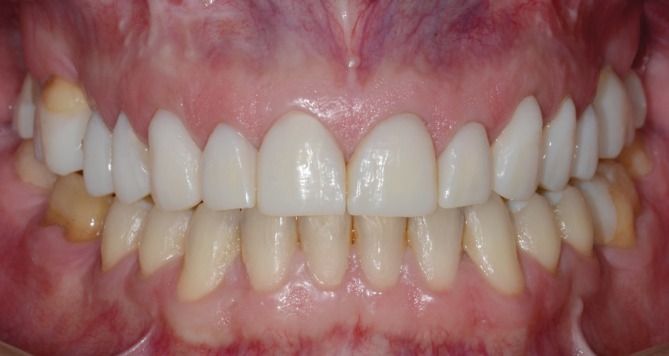
Provisional restorations used during a 2‐month evaluation phase.

**FIGURE 10:**
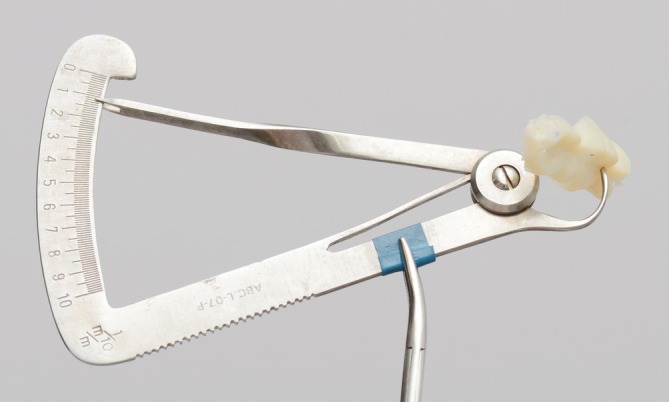
Posterior provisional restorations illustrating the approximate 1.0‐mm thickness of the restorative material.

**FIGURE 11:**
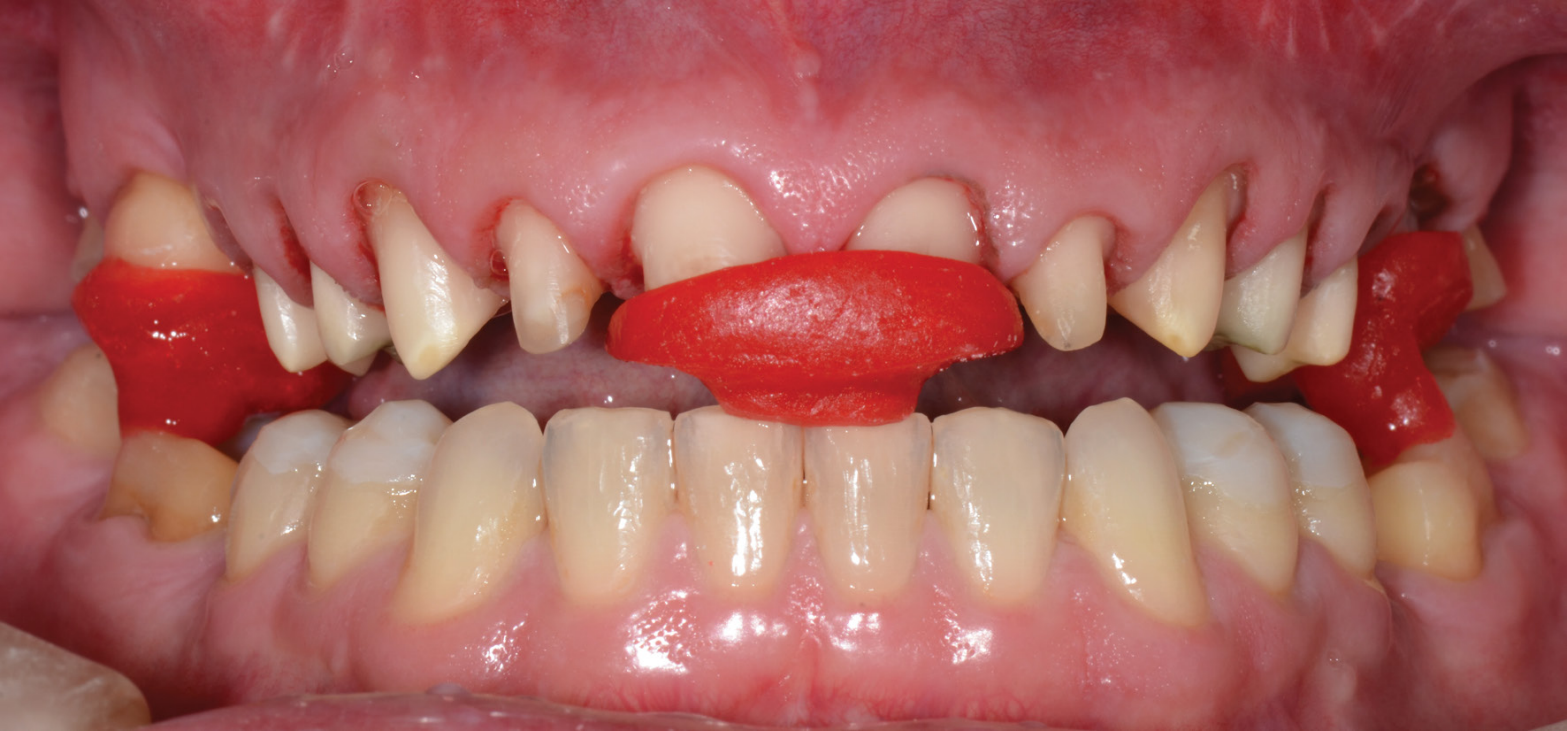
Interocclusal record obtained with acrylic resin at three reference points to preserve the reestablished vertical dimension of occlusion.

**FIGURE 12:**
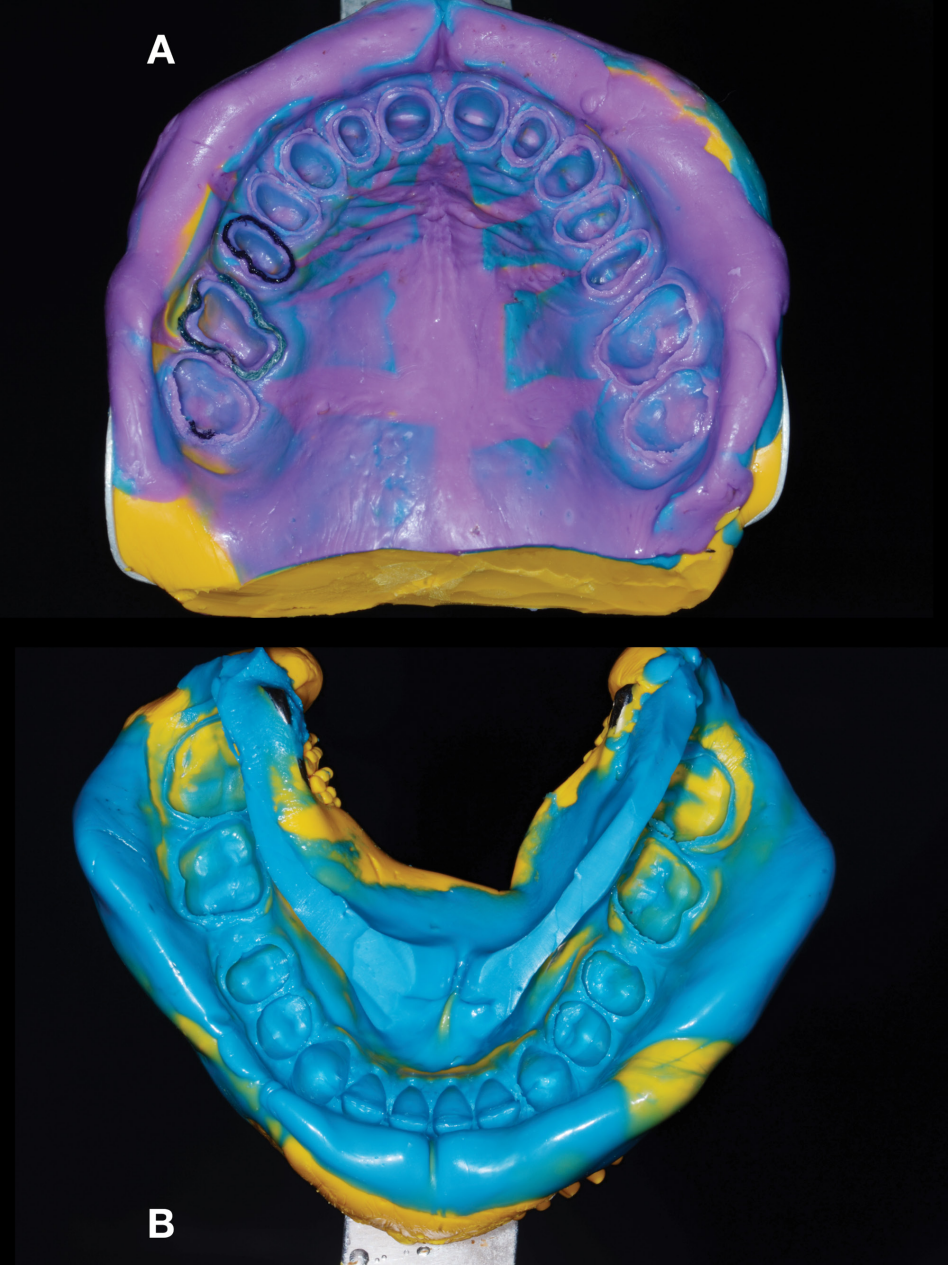
(A) Dental impression for final restorations of the maxillary arch. (B) Dental impression for final restorations of the mandibular arch.

Tooth preparations were performed through the mock‐up (Figures 6, 7, 8) to ensure controlled reduction, further guided by the known diameter of the depth‐cutting bur (cylindrical, rounded tip, 1.2 mm diameter; MF 3098 and flame shape, FG 3118—KG Sorensen, São Paulo, Brazil) for full‐contour restorations with rounded‐shoulder finish lines, while partial restorations were prepared with occlusal anatomical reduction (cone‐shaped, rounded tip, FG 3139—KG Sorensen, São Paulo, Brazil) and a butt‐joint finish line configuration. Additionally, proximal preparation was performed using a cone‐shaped, rounded‐tip bur (FG 2135, KG Sorensen, São Paulo, Brazil), and all internal line angles were rounded in both preparation designs. Finishing and polishing were performed using a standardized grit sequence, progressing from fine‐grit (F) to extra‐fine–grit (FF) burs (MF 3098, FG 3118, FG 3139, FG 2135, KG Sorensen, São Paulo, Brazil), followed by medium‐ and fine‐grit polishers (Jiffy, Ultradent Products, South Jordan, UT, USA). Provisional restorations (Protemp 4, 3M ESPE, Seefeld, Germany) were fabricated to serve as an evaluation phase for 2 months (Figure 9) using the silicone index from the additive wax‐up. For the overlay dental preparations, the immediate dentin sealing was performed on the freshly prepared tooth surface using a total‐etch adhesive system (Adper Scotchbond Multi‐Purpose, 3M ESPE, Saint Paul, MN, USA). Provisional crowns were cemented using temporary cement (Temp‐Bond NE, Kerr, Brea, CA), while overlays were bonded with a flowable resin (Filtek Supreme, 3M ESPE, Saint Paul, MN, USA) (Figure 10), being the dental substrate isolated with petroleum gel. Interocclusal registrations were made using autopolymerizing acrylic resin (GC Pattern resin LS, GC America, Alsip, IL, USA) at three reference points—two posterior and one anterior—to maintain the tested VDO during the provisional phase (Figure 11). Final impressions were obtained using putty‐soft and regular body polyvinyl siloxane (Express XT, 3M ESPE, Saint Paul, MN, USA) for the lower arch, and complemented with light body (Express XT, 3M ESPE, Saint Paul, MN, USA) for the upper arch (Figure 12).

**FIGURE 13:**
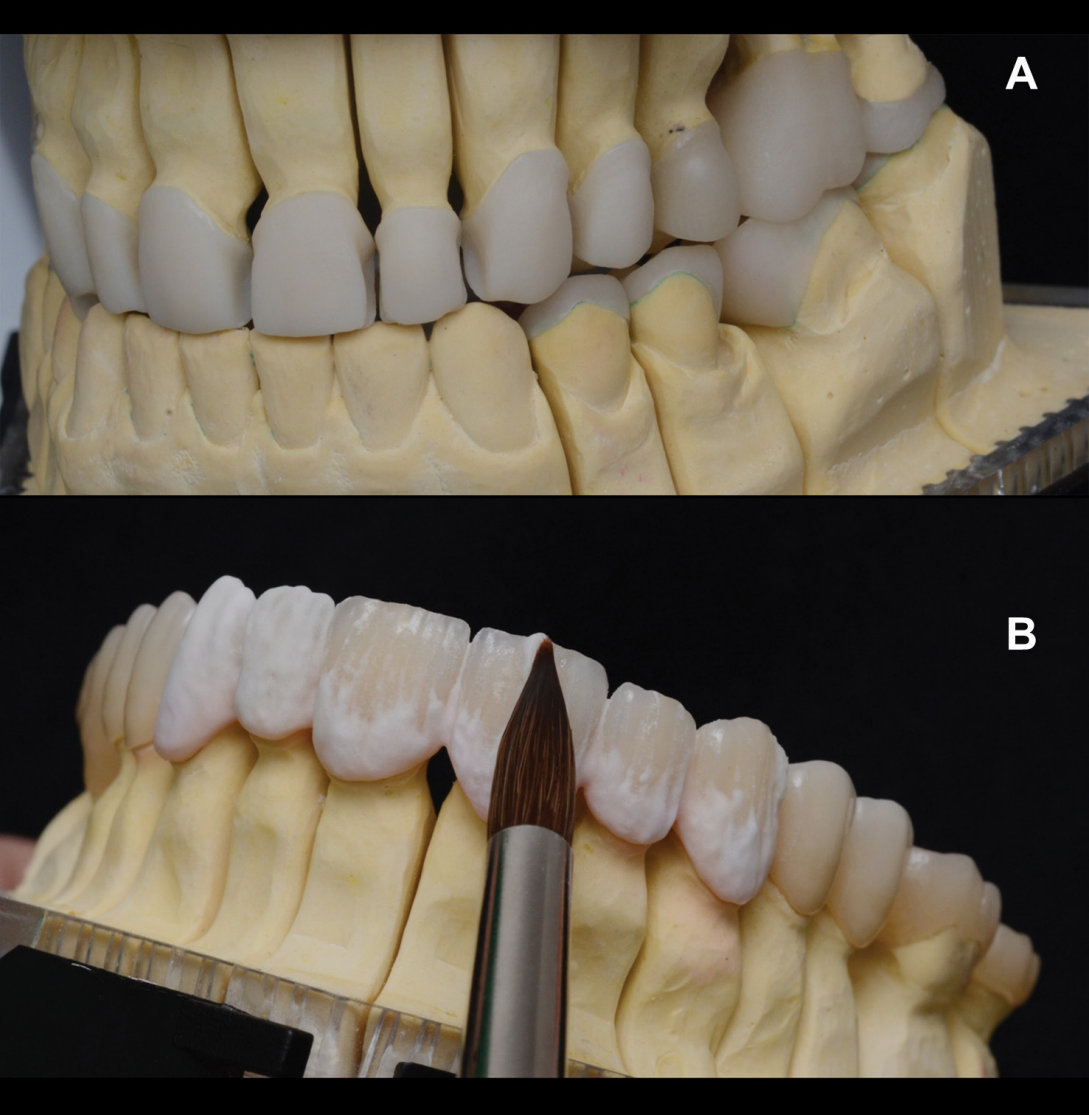
(A) Ceramic restorations with a labial‐occlusal and labial‐incisal cut‐back design. (B) Ceramic layering on the cut‐back regions to enhance esthetic outcome.

**FIGURE 14:**
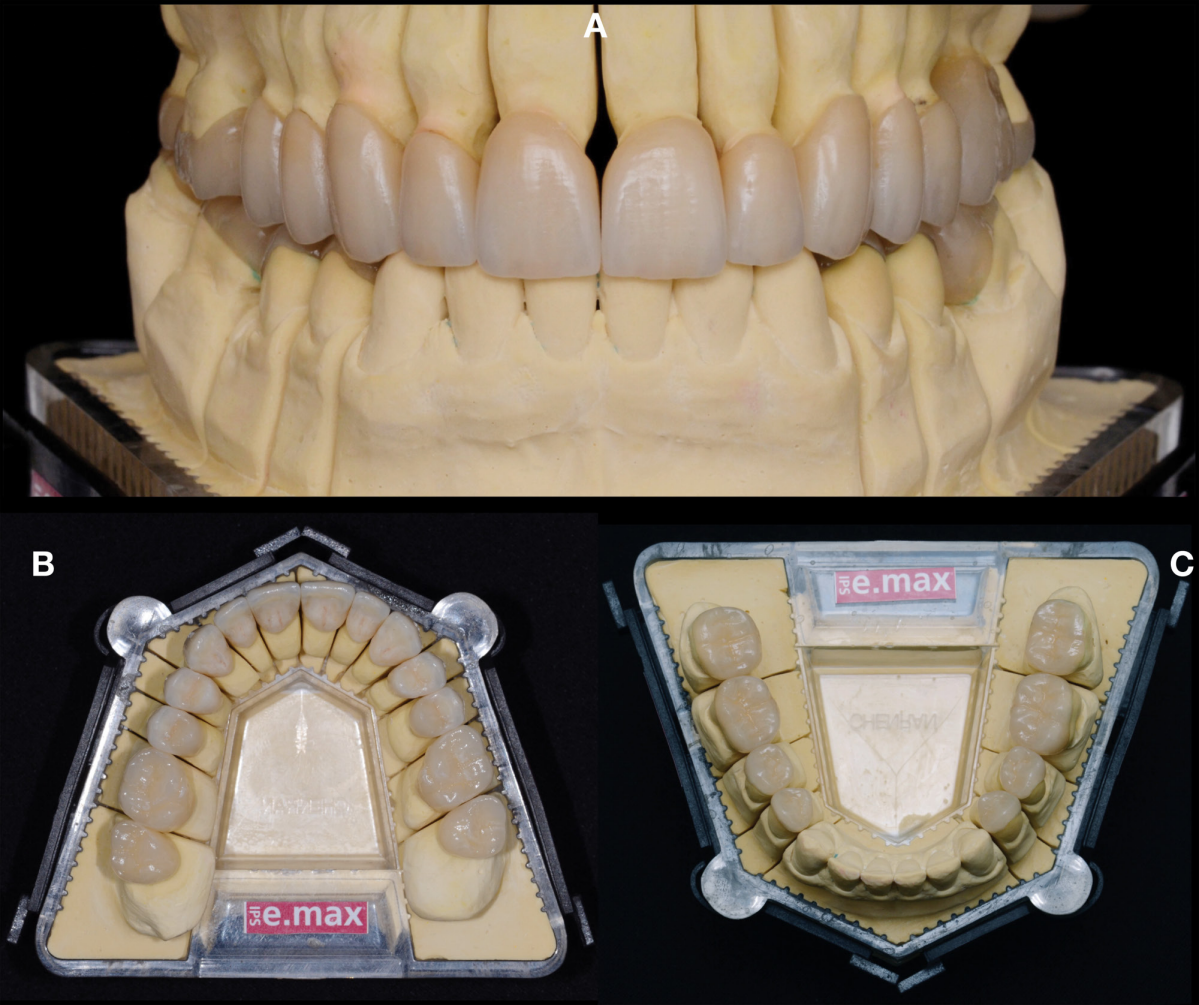
(A) Frontal view of the definitive ceramic restorations. (B) Maxillary occlusal view. (C) Mandibular occlusal view.

Monolithic stained restorations (IPS e.max Press; Ivoclar, Schaan, Liechtenstein) were fabricated for the overlays and crown of the upper molar. Labial‐occlusal and labial‐incisal cut‐backs were performed for the upper premolars and anterior teeth, respectively, to enable porcelain powder/liquid layering (e.max Ceram Ivoclar, Schaan, Liechtenstein) for improved esthetics (Figures 13 and 14).

**FIGURE 15:**
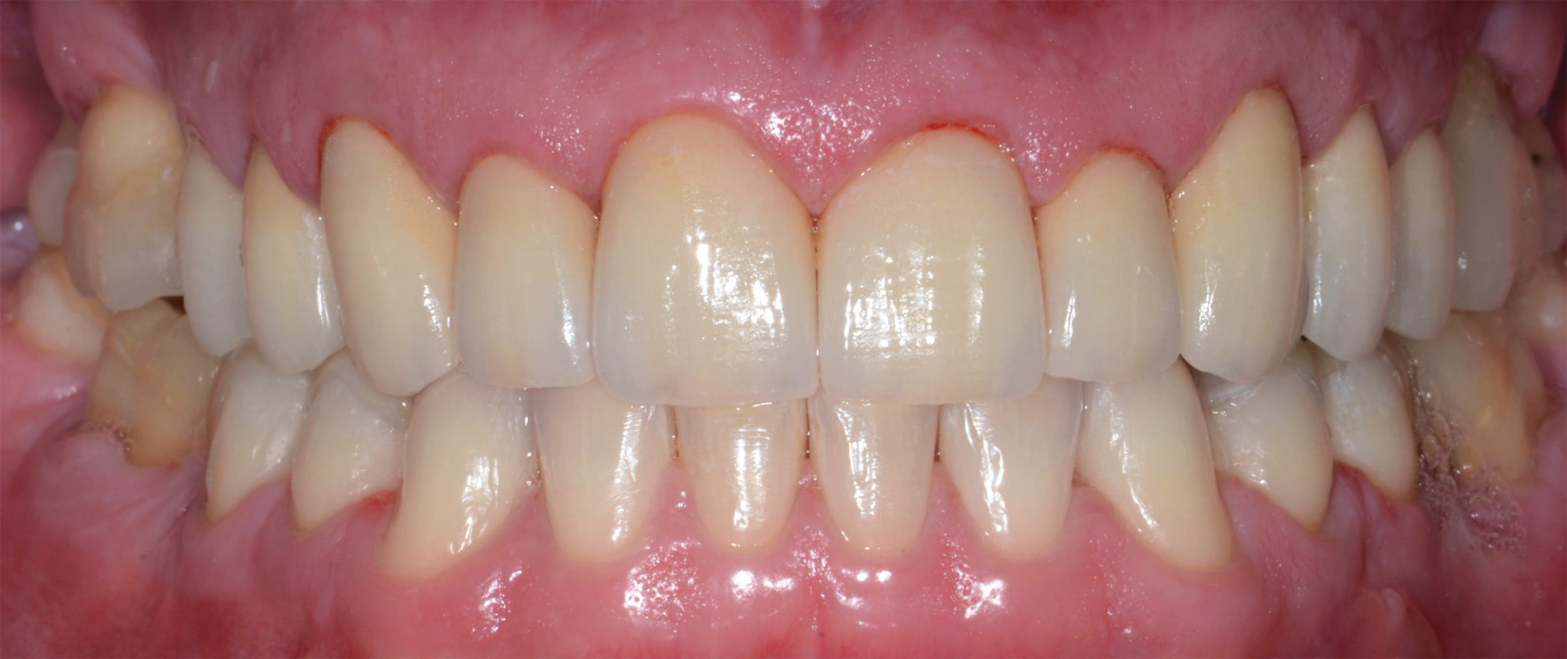
Immediate outcome following the cementation of the ceramic restorations.

Prior to adhesive cementation, dental prophylaxis was performed using pumice paste. Careful relative isolation was achieved with the use of a mouth retractor, cotton rolls, gauze, and high‐volume suction tip, with the assistance of a dental auxiliary. In addition, all the cementation procedures were carried out with six hands to maximize efficiency and to avoid contamination of the operative field. The internal surfaces of all lithium disilicate restorations were etched with 10% hydrofluoric acid (Condac Porcelana, FGM, Joinville, SC, Brazil) for 20 s, thoroughly rinsed with a water‐air spray, and subsequently cleaned with 37% phosphoric acid (Ultra‐Etch, Ultradent Products, South Jordan, UT, USA) for 30 s using microbrush tips to actively clean the vitreous dissolution [38]. A silane coupling agent (RelyX Ceramic Primer, 3M ESPE, Saint Paul, MN, USA) was applied for 60 s. Then, the adhesive (Adper Scotchbond Multi‐Purpose, 3M ESPE, Saint Paul, MN, USA) was applied, air‐thinned for 10 s, and light‐cured (Ultrablue D‐2000; DMC, São Carlos, SP, Brazil) for 20 s. For the overlay preparations, 37% phosphoric acid (Ultra‐Etch, Ultradent Products, South Jordan, UT, USA) was applied for 30 s on enamel and 15 s on dentin, followed by the application of primer and adhesive (Adper Scotchbond Multi‐Purpose, 3M ESPE, Saint Paul, MN, USA) following the manufacturer's instructions. A dual‐cure luting agent (RelyX ARC, 3M ESPE, Saint Paul, MN, USA) was used for overlay cementation, while the crowns were bonded using a self‐adhesive resin cement (RelyX U200, 3M ESPE, Saint Paul, MN, USA). Minimal occlusal adjustments were made using fine‐grit diamond burs (2135FF and 3118FF, KG Sorensen, São Paulo, Brazil), followed by polishing with abrasive rubber points (Ceramisté, Shofu, Kyoto, Japan) (Figure 15). Then, felt discs were used in conjunction with diamond polishing paste (Porcelize, Cosmedent, Chicago, IL, USA) for final surface finishing.

**FIGURE 16:**
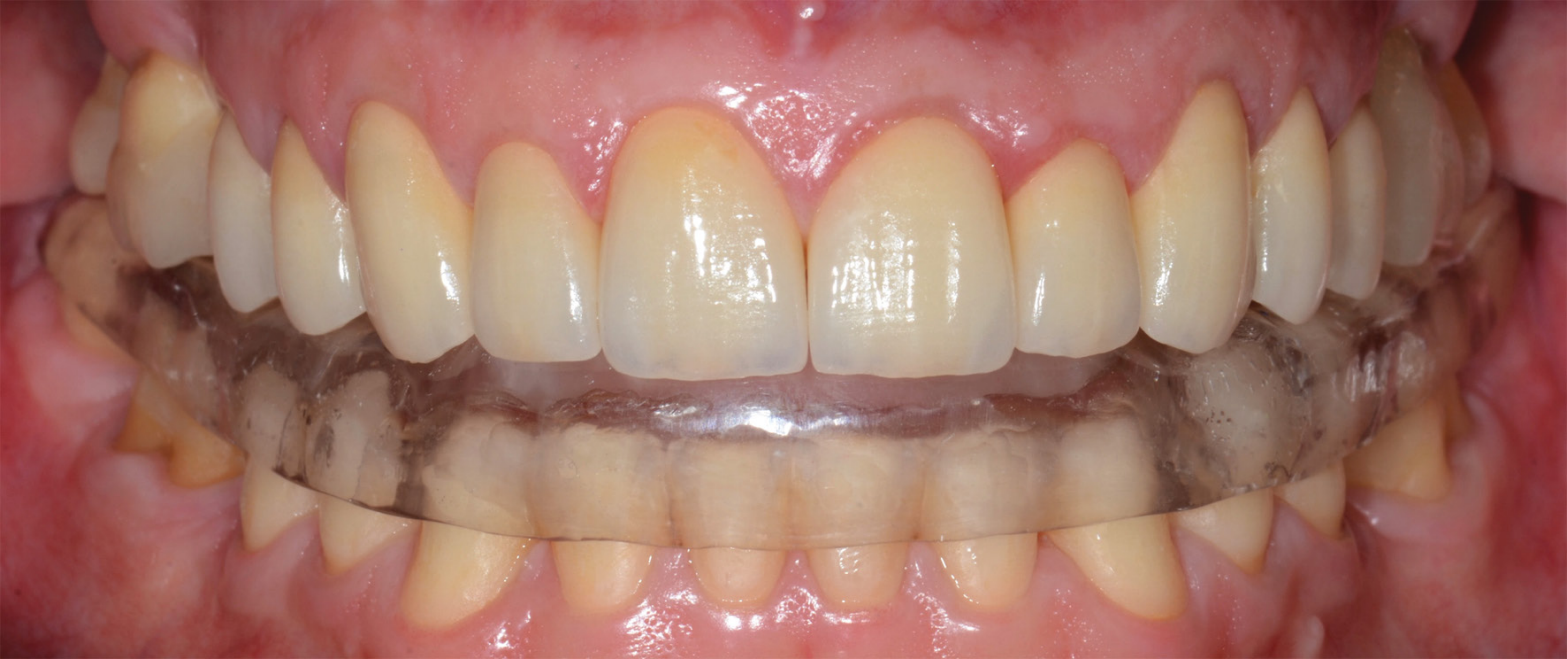
Hard stabilization occlusal splint placed a few days after cementation.

After completing the restorative procedures, a hard stabilization splint for sleep bruxism was fabricated in heat‐cured acrylic resin and adjusted (Figure 16). The patient received specialized treatment for gastroesophageal and psychological conditions. At the postoperative check‐up after cementation, localized swelling was observed in the interdental papilla between the left central and lateral incisors. This condition improved after about 60 days of follow‐up. The suspected cause was residual cement that may have entered the interdental space horizontally, which was not visible on X‐rays or clinically. Another hypothesis is that there was a soft tissue accommodation in response to the horizontal biological distances, to the detriment of the emergence profile of the cemented crowns.

Periodic follow‐ups have been conducted since the cementation procedure. The only complication observed was the debonding of the upper left first premolar crown after 6 years, which was resolved through re‐cementation. The intaglio surface of the crown was cleaned with a sandblaster to remove residual cement, and the internal surface was treated using the same protocol as previously described (hydrofluoric acid, phosphoric acid, and silane coupling agent). Adhesive cementation was carried out with a dual‐cure resin cement (Panavia F, Kuraray Noritake, Tokyo, Japan). The tooth surface was cleaned with pumice paste, and a mixture of equal parts of bottles A and B of the primer (ED Primer II, Kuraray Noritake, Tokyo, Japan) was applied for 20 s and air‐dried for 10 s. Equal amounts of paste A and B of the cement were then mixed, applied to the crown, placed, and light‐cured (Ultrablue D‐2000; DMC, São Carlos, SP, Brazil) using a 20‐s cycle for each surface.

**FIGURE 17:**
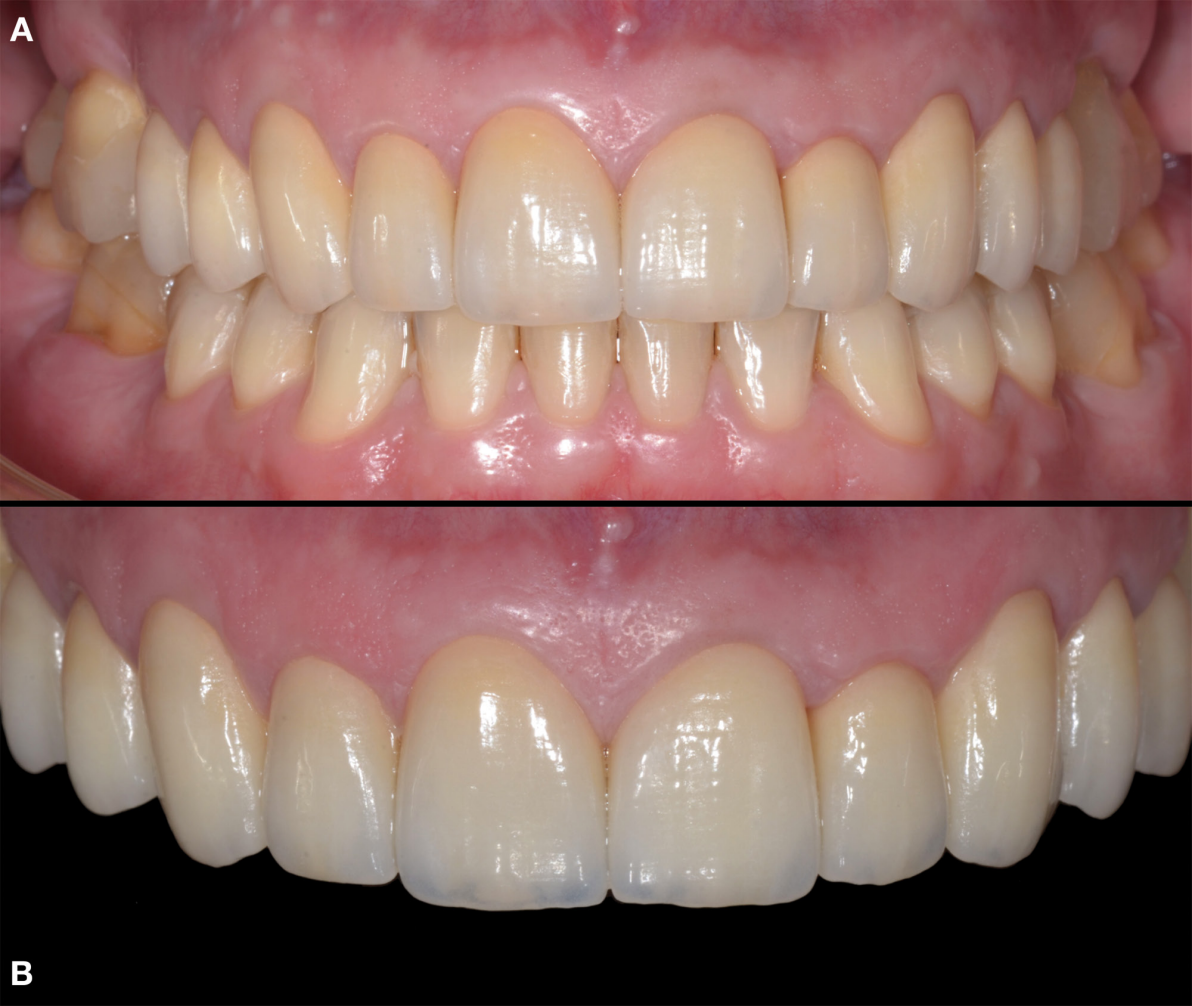
(A) Final outcome of the full‐mouth rehabilitation after soft tissue healing following cementation. (B) Final outcome of the full‐mouth rehabilitation with black background contrast.

**FIGURE 18:**
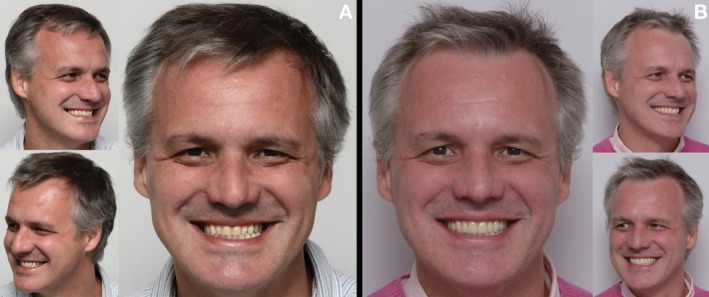
(A) Extraoral views of the patient in the preoperative condition. (B) Extraoral views after a 2‐year follow‐up period.

**FIGURE 19:**
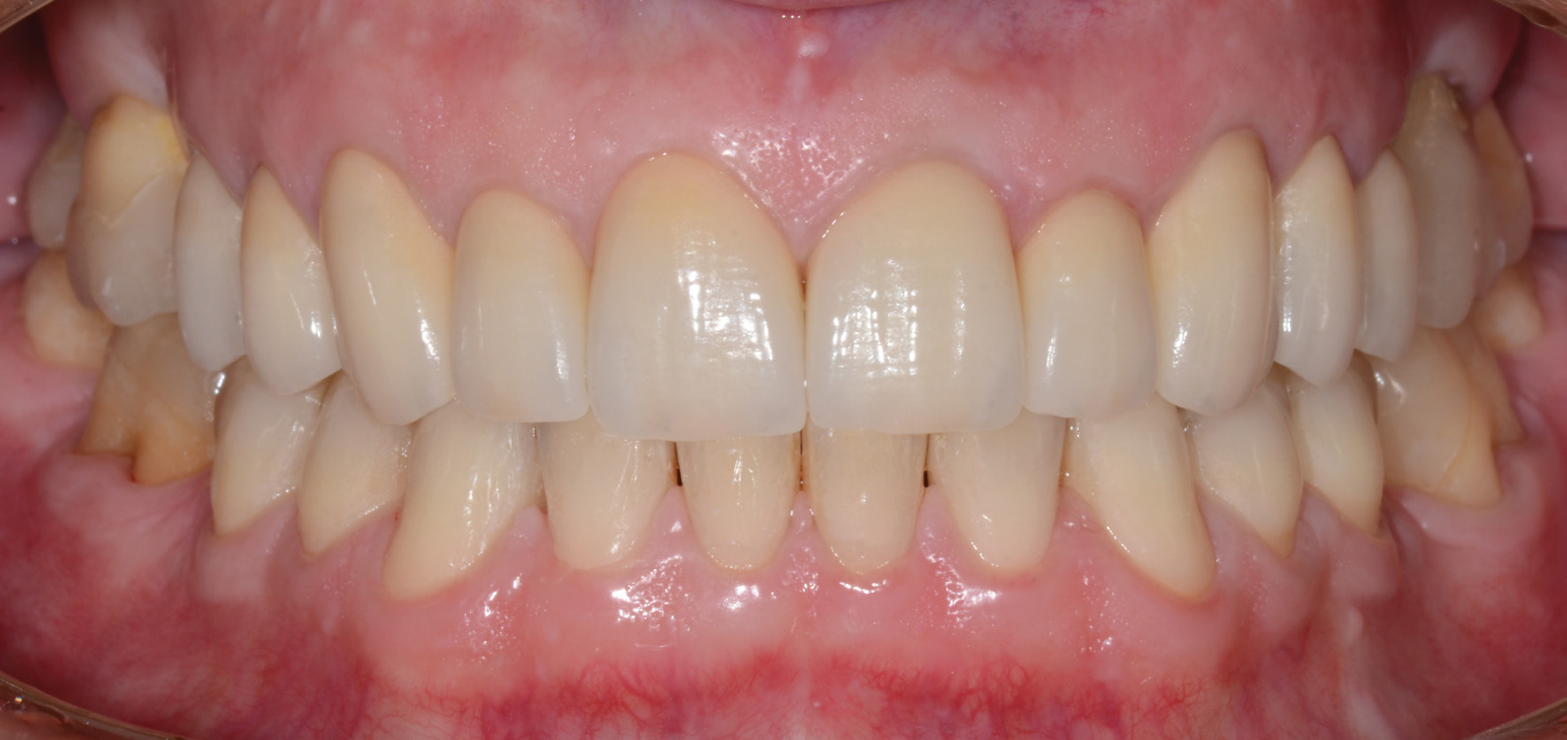
Intraoral frontal view after a 5‐year follow‐up period.

**FIGURE 20:**
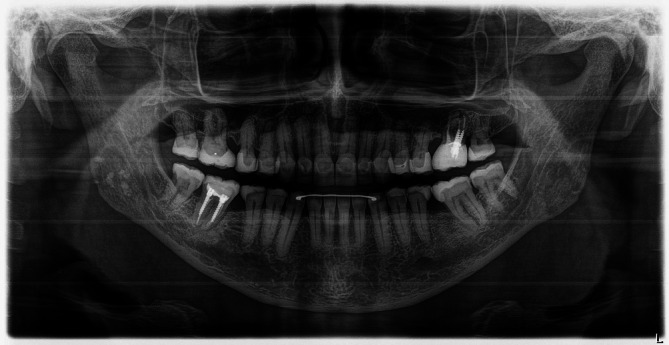
Panoramic radiograph following a clinical follow‐up period of over 11 years.

**FIGURE 21:**
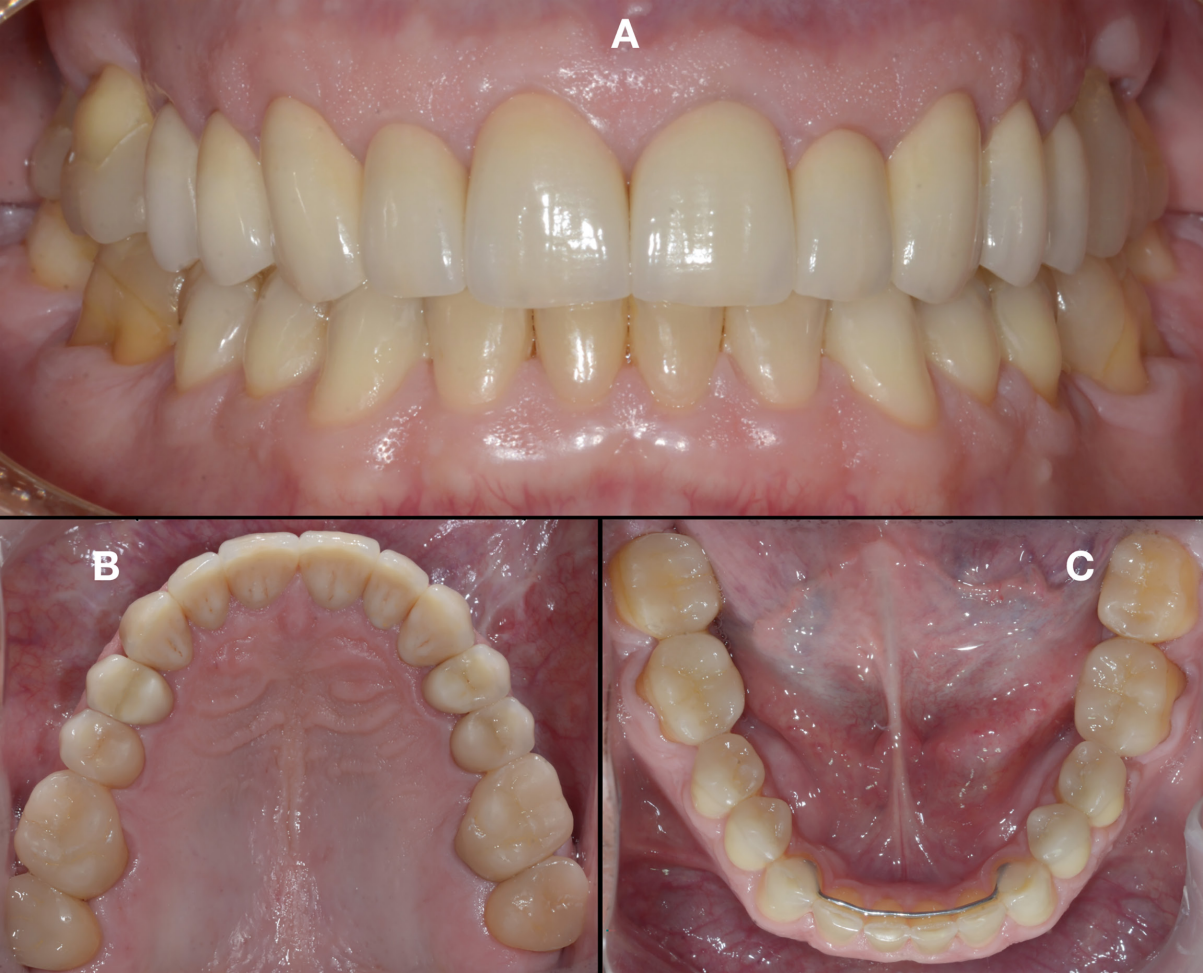
Intraoral views following a follow‐up period of more than 11 years. (A) Frontal. (B) Maxillary occlusal. (C) Mandibular occlusal.

**FIGURE 22:**
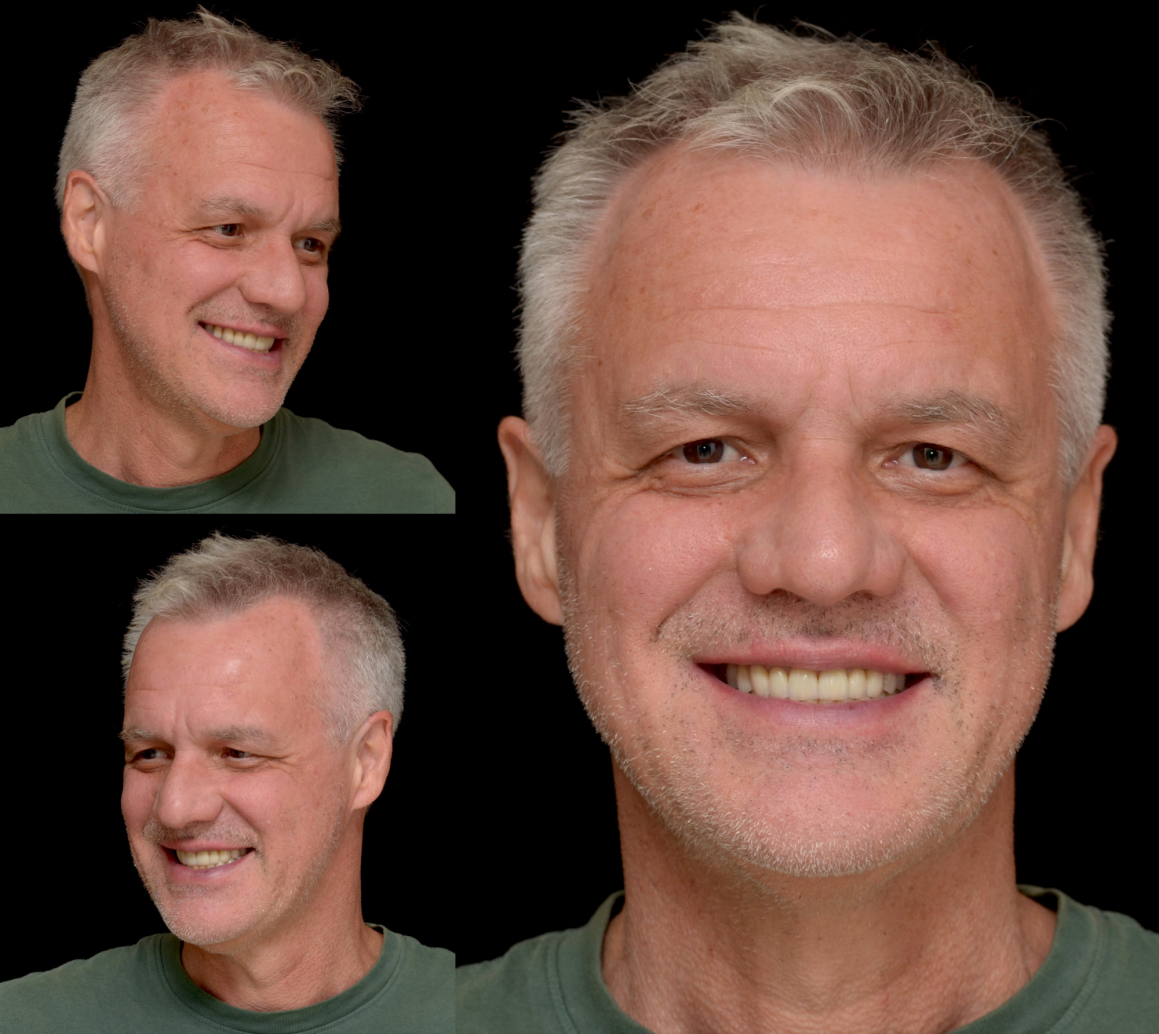
Extraoral views of the patient after more than 11 years of follow‐up.

Following rehabilitation, the VDO was reestablished, and the diastemas were closed, resulting in both esthetic enhancement and functional improvement (Figure 17). Furthermore, dentin hypersensitivity was successfully treated, as the patient reported no symptoms. Medium‐term follow‐ups are depicted in Figures 18 and 19. The last follow‐up visit was in October 2025. Clinically, no marginal gaps, signs of degradation, or evident/significant discoloration were observed. The occlusal, palatal/lingual, and incisal surfaces of the restorations showed no wear patterns, with only a slight loss of surface gloss observed on the occlusal areas. The buccal surfaces remained esthetically pleasing, exhibiting adequate gloss and color stability. The natural opposing mandibular anterior teeth displayed shallow incisal wear that was not substantially different from the pattern documented 11 years earlier (Figures 20, 21, 22). The outcomes have remained stable over the 11+‐year follow‐up period, with the patient consistently reporting comfort, no functional issues, and satisfaction with the esthetic result. The biological, mechanical, functional, and esthetic stability are credited not only to the restorative treatment but also to the effective management of parafunctional habits and multiple etiological factors involved.

## 3 Discussion

Tooth wear is becoming more common in younger patients, highlighting the importance of preventive measures, early detection, and interceptive treatment for dental professionals to avoid the need for extensive full‐mouth rehabilitation [13]. When preventive and interceptive measures are not applied at the appropriate stage, the adequate execution of restorative treatment is essential to ensure the long‐term success of severe tooth wear cases [14]. Achieving this goal also depends on accurately diagnosing and managing the underlying causes of this condition.

Several restorative approaches have been described, employing different techniques and materials, primarily resin‐based composites and ceramics [15, 16, 17, 18, 19, 20]. In this case, the decision to use an indirect approach with lithium disilicate partial and full‐contour restorations was based on the patient's age, the extent of tooth structure loss involving both buccal and lingual surfaces, and the presence of defective existing restorations [14]. Also, a mixed approach combining palatal resin‐based composites with facial ceramic veneers may represent a viable alternative, particularly for the upper anterior teeth [17, 18, 19, 20]. However, due to the extensive palatal wear, which closely resembled a finish line, a full‐contour restoration was indicated, requiring minimal intervention in that area aside from refining the margin. Additionally, the use of monolithic restorations reduces technique sensitivity, provides uniform material thickness, and maintains a single adhesive interface (lithium disilicate–tooth substrate). In contrast, a mixed approach increases the number of adhesive interfaces (lithium disilicate–tooth substrate and lithium disilicate–resin‐based composite), thereby elevating technique sensitivity.

Resin‐based composites and ceramics are the most commonly used esthetic restorative materials in dentistry [28]. Composites offer the advantage of a direct and more conservative approach, along with easy reparability [13, 15, 21]. Additionally, the CAD/CAM technology allows for the fabrication of thin restorations that can be placed with minimal or without tooth reduction [20, 35]. In contrast, ceramics require an indirect technique, which is generally more invasive [14]. However, they provide greater resistance to fracture, surface degradation, and better long‐term retention of surface gloss, offering superior performance over time compared to composites, which typically require more frequent maintenance [28, 32, 35].

The rehabilitation of severe tooth wear is often challenging due to the presence of excessive mechanical forces and biocorrosion in the oral environment. Factors such as sleep and awake bruxism, gastroesophageal reflux, and unfavorable dietary habits contribute to these conditions and may accelerate the degradation of restorative materials [23]. While the identification and management of etiological factors are essential, the longevity of restorations also depends on their ability to withstand adverse functional conditions without compromising their structural and esthetic properties. In vitro studies [24, 25] have shown that resin‐based composites undergo accelerated degradation in acidic environments, leading to reduced mechanical properties and increased surface roughness [24, 25]. This deterioration is primarily attributed to the hydrolysis of the matrix and coupling agent [24]. Similarly, ceramics are also susceptible to acidic degradation, resulting in changes in gloss, color, and translucency [26, 27]. For lithium disilicate, degradation occurs primarily through surface roughening caused by crystals dissolution from the glassy matrix [27].

Clinical research has identified lithium disilicate as the most commonly used ceramic for rehabilitating severe tooth wear, a preference attributed to its favorable combination of mechanical strength and esthetic properties [29]. Numerous clinical trials have demonstrated that lithium disilicate partial and full‐contour restorations in the anterior and posterior regions are reliable and durable in complex restorative scenarios [30, 31, 32, 33, 34, 35, 36]. However, even among studies [32, 33, 34, 35, 36] reporting high survival rates, some shortcomings were noted. The majority of these studies employed a modified version of the US Public Health Service (USPHS) criteria [32, 33, 34, 35, 36] for clinical evaluation, using ratings such as Alpha (no problems observed), Bravo (minor complications), and Charlie (major complications requiring restoration replacement). Minor complications (Bravo), including small chippings, marginal discoloration, crack formation, loss of retention, occlusal wear, and endodontic issues, were reported and could often be managed with intraoral repair, reattachment, or monitoring [32, 33, 34, 35]. In contrast, one study [36] reported major complications (Charlie), such as fractures, debonding, and loss of occlusal contact, which required the replacement of the restoration.

The presented restorative treatment demonstrated favorable stability for more than 11 years, with no evidence of significant marginal discoloration, secondary caries, fractures, or wear, and only a slight loss of surface gloss on the occlusal areas, which may be attributed to the loss of the superficial glaze/staining layer and surface roughening caused by crystal dissolution [27]. The buccal surfaces exhibited adequate gloss and color stability. As previously mentioned, the only major complication observed was the debonding of an upper premolar crown, which was easily resolved through re‐cementation. The ceramic strategy employed consisted of a labial cut‐back design for layering on the upper anterior and premolar teeth, along with a stained monolithic approach on the functional cusps of the upper premolars and the other posterior restorations. This approach has been validated in a prospective study reporting an annual failure rate of just 0.5% over follow‐up periods of up to 13 years [32]. Furthermore, another prospective study [34] evaluating posterior restorations with a minimum thickness of 1.0 mm provided clinical data up to 11 years, reinforcing the reliability and long‐term success of this treatment approach.

The decision‐making process regarding techniques and materials primarily depends on the extent of tooth structure loss. Resin‐based composites are often preferred for interceptive or moderate cases due to their conservative nature [14]. However, in cases of severe tooth wear, ceramics—particularly lithium disilicate—often emerge as the material of choice. An in vitro study [27] comparing zirconia and lithium disilicate demonstrated greater stability in translucency and color for zirconia under acidic conditions. Nevertheless, a notable baseline difference in translucency between the two materials was observed [27]. A randomized controlled clinical trial [33] compared the prosthetic rehabilitation of extensive tooth wear using lithium disilicate and translucent zirconia crowns. The study found that both materials performed similarly, but lithium disilicate crowns were rated as more esthetically pleasing, even when translucent zirconia was veneered with porcelain.

In the present clinical case, the VDO was reestablished using a Lucia JIG to obtain an intermaxillary record for model mounting in a semi‐adjustable articulator and additive diagnostic wax‐up. Similarly, other studies [32, 34] have also reported wax‐ups at centric relation. Some authors [31] mounted casts using an arbitrary facebow and posterior wax record, while others [17, 18, 19] started with an anterior and premolar wax‐up to evaluate incisal edge position and occlusal plane, followed by a posterior wax‐up. Another study [15] recorded the intermaxillary relationship based on the myocentric position and the minimal space required to restore lost tooth structure. As shown, there are no clear guidelines for a specific, unanimous, and unquestionable technique to reestablish VDO, as the various approaches have yielded favorable results [11]. Likewise, the evaluation phase with provisional restorations varies in duration among different studies [12]. The present case used a 2‐month period, while other studies [32, 34] ranged from 3 months or more. Some authors [31] omitted this phase, and others [17, 18, 19] extended it further.

Certain factors usually considered critical in tooth wear rehabilitation become less significant when the diagnosis is accurate, the indication is appropriate, the technique is properly executed, and when strict postoperative follow‐up is consistently maintained. It is essential to emphasize that restorative treatment restores esthetics and function, but it does not address the underlying causes of tooth wear associated with early oral aging syndrome [6]. Therefore, an interdisciplinary approach is required. In this case, psychological and gastroesophageal management were used to address the etiological factors, along with parafunctional control using a hard occlusal splint. A well‐established diagnosis, combined with comprehensive treatment planning that includes VDO reestablishment techniques, appropriate restorative material selection, and effective management of etiological factors, was key elements contributing to the success of the present case. These principles may serve as a valuable clinical guide for dental professionals facing the challenges of managing severe tooth wear in daily practice.

Additionally, a comprehensive medical history and thorough clinical examination are essential for detecting initial signs of early oral aging syndrome, enabling the implementation of interceptive and preventive measures to reduce the need for extensive full‐mouth rehabilitation. Young patients presenting with dentin hypersensitivity, enamel fracture lines, and mild tooth wear—especially when associated with anxiety, gastroesophageal reflux, acidic dietary habits, or sleep bruxism—may fall within a zone of increased susceptibility [6, 7]. Greater awareness of this condition should be promoted among dental professionals to support early diagnosis and intervention.

## 4 Conclusions

Within the limitations of this case report, the full‐mouth rehabilitation using partial and full‐contour lithium disilicate restorations for severe tooth wear demonstrated a favorable outcome, with an 11+ year follow‐up confirming its appropriate mechanical behavior, maintenance of esthetic outcomes and preservation of functional integrity, addressing the complex multifactorial etiological factors associated with tooth wear. The long‐term success of this rehabilitation emphasizes the importance of a comprehensive, interdisciplinary approach, incorporating careful treatment planning and proper management of underlying etiological factors for sustained clinical performance.

## Funding

The authors have nothing to report.

## Disclosure

The authors do not have any financial interest in the companies whose materials are included in this article.

## Conflicts of Interest

The authors declare no conflicts of interest.

## Data Availability

The data that support the findings of this study are available on request from the corresponding author. The data are not publicly available due to privacy or ethical restrictions.
